# Lactotrophs: The new and major source for VEGF secretion and the influence of ECM on rat pituitary function *in vitro*

**DOI:** 10.3892/or.2015.3851

**Published:** 2015-03-13

**Authors:** JOACHIM ALFER, JOSEPH NEULEN, ANDREAS GAUMANN

**Affiliations:** 1 Department of Pathology Kaufbeuren-Ravensburg, D-88212 Ravensburg, Germany; 2 Department of Gynecological Endocrinology and Reproductive Medicine, RWTH University of Aachen, School of Medicine, D-52057 Aachen, Germany

**Keywords:** pituitary, lactotrophs, folliculostellate cells, VEGF, cell culture, extracellular matrix

## Abstract

Vascular endothelial growth factor (VEGF) plays a pivotal role in pituitary endocrine function by influencing fenestration and blood vessel growth. Folliculostellate (FS) cells, which represent only a small number of pituitary cells, are recognized to produce VEGF. Tissue sections and primary pituitary cell cultures from rat pituitary glands were performed to co-localize VEGF and pituitary lactotrophs, which represents nearly 50% of all pituitary cells, by immunofluorescence. VEGF is co-localized with prolactin-producing cells *in vivo* and *in vitro*. FS cells are present infrequently *in vivo* (1.6%) and *in vitro* (2.4%). Culture supernatants were analyzed for the presence of VEGF by ELISA. VEGF levels are always significantly lower in supernatants from the cells that are seeded on Matrigel extracellular matrix (ECM) compared to the cells grown on plastic. Lower VEGF concentrations in supernatants from the pituitary cells cultured on ECM may reflect a more adequate cell environment compared to culture on plastic. These results demonstrate for the first time, that VEGF is expressed by lactotrophs, which outnumber FS cells. These results are of potential clinical relevance especially in oncology for the interpretation of studies investigating anti-angiogenic treatment of pituitary tumors.

## Introduction

Folliculostellate (FS) cells are the first cell type in which vascular endothelial growth factor (VEGF) production was detected. Until now, FS are believed to be the only cell population within the pituitary gland to produce VEGF (1,2). S-100 is a reliable marker for FS cells (3). Vankelecom *et al* (4) described that ~7.5% of cultured mouse pituitary cells were stained for the presence of S-100. It has been suggested that FS cells perform several supportive functions. They are involved in the regulation of phagocytosis (5), and produce various growth factors (6,7) and cytokines (4,8). Recent studies suggest that they represent pluripotent adult stem cells (9). *In situ* hybridization has demonstrated homogeneous signals of VEGF mRNA in rat pituitary, with an upregulation of VEGF expression in estrogen-treated animals (10). Western blot analysis and RT-PCR of anterior pituitary tissues showed the presence of VEGF164 and VEGF120 (10). Jabbour *et al* (11) described that only scattered positive FS cells are present within the sheep pituitary. They identified 20% that were double-stained cells for VEGF and S-100; ~80% of cells that were stained only positive for VEGF, but were unidentified.

It is well known that extracellular matrix (ECM) plays an important role in studying cellular function *in vitro*. In the physiological environment, like ECM, cytokines and hormones, are absent under culture conditions of the pituitary cells. Laminine and type IV collagen are typical ECM constituents in both the epithelial and vascular basement membrane of the pituitary gland (12,13). The ECM components are able to influence the morphological appearance, growth behavior and migratory activity of epithelial cells. Both, tumor and normal cells of epithelial origin (lactotrophs), are more likely to reflect their *in vivo* counterparts when maintained on ECM, instead of uncoated plastic (14). ECM components also influence pituitary function *in vivo* (15,16).

The present study was designed to identify VEGF producing cells, other than FS cells, in the anterior pituitary gland of rats *in vivo* and *in vitro* and to investigate the influence of ECM (Matrigel) on VEGF production in primary pituitary cell cultures.

## Materials and methods

### Ethics statement

The present study was performed in accordance with the recommendations of the Guide for the Care and Use of Laboratory Animals of the National Institutes of Health. The protocol was approved by the local Ethics Committee for animal experiments of RWTH University of Aachen, School of Medicine (permit no. 1077 A4; 2001). All the experiments were performed without suffering of the animals.

### Animals

Adult female Sprague Dawley rats of 250 g body weight were used, bred from a colony at the Institute for animal research (RWTH University of Aachen, Germany). Rats were housed in groups of four per cage in a quiet and light-regulated room; the temperature was maintained at 20–21°C. Food and water supply was available *ad libitum*. Experiments were started between 9–10 h to exclude changes associated with circadian rhythm. Vaginal swabs were taken at 8 h, were alcohol fixed and stained (Papanicolaou). Rats at the estrous stage were sacrificed by cervical dislocation under anesthesia with Forene (Isofluran, Abbot). The pituitary was removed immediately and formalin-fixed for paraffin-embedding, deep-frozen or treated for cell-culture/cytospin-preparation as described later.

### Materials

Cell culture materials and reagents were obtained from Greiner Labortechnik (Solingen, Germany), Falcon (Heidelberg, Germany), Seromed (Berlin, Germany) and Sigma (Deisenhofen, Germany). Matrigel was obtained from Becton-Dickinson (Heidelberg, Germany). A list of antibodies is given in Table I.

### Cell culture

Rat pituitary monolayer cell cultures were generated following a modified protocol (17). In brief, the pituitaries were washed 3 times and then cut into small pieces.

Cell dissociation was performed with trypsin-type III (25 mg/5 ml medium) for 15 min at 37°C. DNase type V solution (10 mg/5 ml medium) was added for 1 min. After centrifugation at 500 g for 2 min, supernatants were removed and cell pellets were incubated for 5 min with a trypsin inhibitor (type I-S, 5 mg/5 ml medium) and again centrifuged for 2 min. The cells were then incubated in EDTA solution, 2×5 min in 2 mM EDTA and 2×5 min in 1 mM EDTA. The digested pieces of the pituitary were dispersed and cell viability was determined by trypan blue exclusion. After counting, the cells were seeded into 24-well plates (TPP tissue culture plates, Sigma) at a concentration of 300,000 cells/well. Cells were incubated at 37°C and 5% CO_2_ in medium 199 with 2.2 g/l NaHCO_3_ supplemented with 10% FCS (inactivated for 1 h at 56°C), 2 mM glutamine and penicillin/streptomycin (10^5^ U/10^5^ *μ*g/ml). After 2 days the medium was changed. On day 4, monolayers were washed with PBS and serum-free culture medium was added for 24 h to dilute the serum. On day 5 serum free media were replaced for another 48-h incubation period.

### Culture on Matrigel and plastic

Accordingly, cells were cultivated on Matrigel (Becton-Dickinson). Matrigel was diluted 1:1 with a serum-free medium. Culture plates were covered with Matrigel solutions and incubated for 1 h at 37°C for polymerization. Cells were seeded on top of the gel. Supernatants from Matrigel-coated wells as well as from plastic, were pooled (12-wells/culture), centrifuged and stored at −20°C for VEGF quantification.

### Immunohistochemistry

Immunohistochemistry was performed on formalin fixed (fixation with buffered formalin, pH 6.5–7.2) paraffin-embedded pituitary gland sections (4 *μ*m) and cryostat sections of resected pituitary glands (6 *μ*m). A streptavidin-biotin-peroxidase method was employed. Paraffin sections were deparaffinized and rehydrated in PBS. Cryosections were fixed in acetone (4°C for 10 min). Cell cultures were fixed in methanol (4°C for 10 min). Endogenous peroxidase activity was blocked with 0.3% hydrogen peroxide for 30 min. Paraffin sections were incubated with trypsin (Dako, Hamburg, Germany) for 15 min. For negative controls, phosphate-buffered saline (PBS; Dulbecco) diluent, without Ca^2+^ and Mg^2+^, containing 1.5% bovine serum albumin (BSA) replaced the primary antibody. In addition, rabbit immunoglobulin G (IgG; Dako), goat IgG (Dianova, Hamburg, Germany) and normal mouse IgG (Dianova) were used at the identical concentration as the primary antibody. Human endometrium of mid-luteal phase, from a patient of proven fertility, served as a positive control for VEGF staining. Endometrial tissue block was used with the informed consent of the participant patient (18).

Primary antibodies (Table I) were applied overnight at 4°C for paraffin embedded sections, whereas cryosections, cytospin-preparations of pituitary cell suspension (detection of lactotroph and FS cells) and cultured cells were incubated for 4 h. We used the LSAB2-kit (kit for use on rat specimens, Dako) for primary mouse and rabbit antibodies. Visualization of specific antigens was performed by peroxidase-catalyzing substrate and converting with chromogene aminoethyl carbazole (AEC; Zymed Laboratories Inc., San Francisco, CA, USA) to a red-colored deposit.

### Double staining of VEGF and prolactin

Staining was performed on 24-well culture plates. After removing culture media, cells were fixed with methanol (4°C for 10 min), rehydrated with PBS and blocked with a donkey serum (dilution 1:20 in PBS), followed by an overnight incubation with prolactin antibodies (dilution 1:30 in PBS/2% rat serum, Santa Cruz, CA, USA) at 4°C. The secondary antibody (TRITC, donkey anti-goat, Dianova) was diluted 1:100 in PBS (pH 8.2) containing a 2% rat serum and was incubated for 30 min. After three rinses with PBS, the cells were blocked with goat serum (dilution 1:20 in PBS, 10 min), followed by incubation with an anti-VEGF-A antibody (dilution 1:30 in PBS/2% rat serum, Santa Cruz) overnight at 4°C. The secondary antibody (FITC, goat anti rabbit, Linaris) was diluted 1:50 in PBS (pH 8.2+2% rat serum) for 30 min. The cells were incubated with KCl (200 mmol) for 5 min and then with DAPI (0.2 *μ*g/ml PBS pH 7.0, Sigma) for 1 h and finally mounted with medium for fluorescence analysis (Vector Laboratories, Burlingame, CA, USA).

For negative controls phosphate-buffered saline (PBS, Dulbecco, w/o Ca^++^, w/o Mg^++^) containing 2% rat serum replaced the primary antibody. Rabbit IgG (Dako) and goat IgG (Dianova) were applied at equal concentrations as the primary antibody.

### VEGF detection within culture medium

VEGF was quantified by ELISA (R&D Systems, Wiesbaden-Nordenstadt, Germany; test validations according to the provider’s specification), which is able to identify the VEGF-164 and VEGF-120 isoform of the mouse. Cross-reactivity (>95%) to the corresponding rat VEGF enables the application of this ELISA. Protein detection is done by the Lowry-test.

### Identification of FS

FS cells, identified by monoclonal and polyclonal antibodies against S-100 protein (Table I), were counted on cytospin-preparations of dispersed pituitary cells, after seven days of culture and on cryostat sections as well as on formalin-fixed and paraffin-embedded sections of the pituitary gland.

### Estimation of cell type distribution

For semi-quantification analysis of the different cell types 10 high-power fields were analyzed.

### Statistical analysis

Experiments were repeated six times. The Mann-Whitney U test was used for statistics. Data are shown as means ± standard deviation.

Illustrations were performed with Axiovert 135 microscope (Zeiss, Göttingen, Germany). We performed quadruple overlay of immunohistochemical analysis as follows: i) immunocytochemical detection of prolactin; ii) immunocytochemical detection of VEGF; iii) DAPI counterstaining; iv) light microscopic picture.

## Results

### FS in vivo and in vitro

**Figure. 1:**
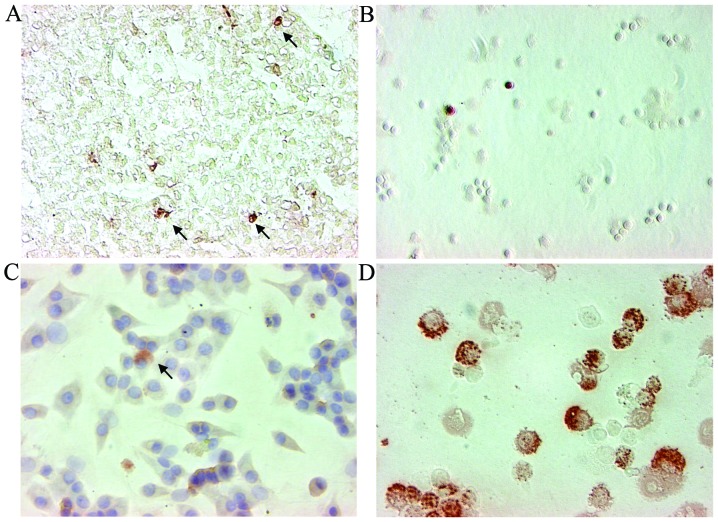
(A–C) Folliculostellate cells *in vivo* and *in vitro*. (A) Cryostat section (4 *μ*m) of rat anterior pituitary gland by immunohistochemistry with S100 antibody. Scattered cells are stained as indicated by the black arrows. (B) Dispersed pituitary cells after treatment with collagenase before seeding into wells (cytospin-preparation). Only scattered cells are immunocytochemical positive for S-100 indicated by the black arrows. (C) Primary pituitary cells after 7 days of culture on plastic. A single S100 positive cell is detected as indicated by the black arrow. The cells are counterstained with hematoxylin. (D) Dispersed pituitary cells after treatment with collagenase before seeding into wells (cytospin-preparation). Nearly 50% of the cells are immunocytochemically positive for prolactin.

FS cells are identified by two different antibodies against S-100 protein. Both antibodies, monoclonal (BioGenex, Hamburg, Germany) and polyclonal (Dako), showed similar results. S-100 positive FS cells are infrequently detected in cryostat (Fig. 1A) and paraffin sections (data not shown) of the anterior pituitary gland. Approximately 1.6% of the pituitary cells stain positive for S-100. We also performed a cytospin-preparation of dispersed pituitary cells (before seeding into culture wells) to identify S-100 positive cells by immunocytochemistry. Only few FS were identified (Fig. 1B), even after seven days of culture only the scattered stained cells were visible (2.4% S-100 positive cells; Fig. 1C).

### VEGF and prolactin in vivo and in vitro

**Figure. 2:**
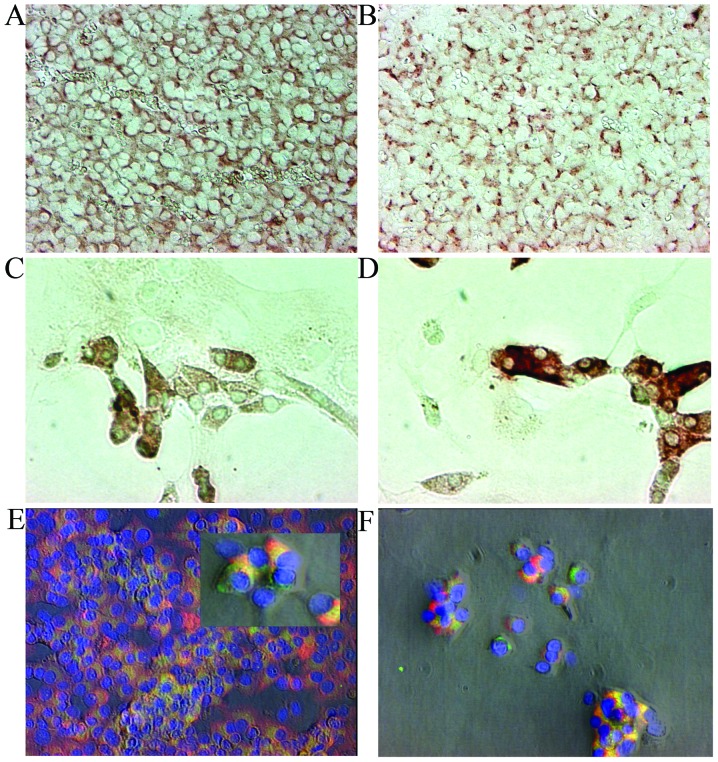
Paraffin serial sections and cell cultures with immunostaining for VEGF and prolactin. (A) Paraffin section of anterior pituitary gland with an even signal for VEGF (red deposit, AEC). (B) Paraffin section with nearly identical signals for prolactin (red deposit, AEC), when compared with VEGF. (C and D) cultures of primary rat pituitary cells on plastic dishes after 7 days of culture demonstrate positive immunocytochemical signals for VEGF (C) and prolactin (D). (E and F) Cultures of primary rat pituitary cells on Matrigel (E) and plastic dishes (F) after 7 days of culture. (E) Double staining for prolactin (TRITC, red) and VEGF (FITC, green). Many cells stain for prolactin and VEGF (yellow). Negative and single stained cells are seen. The inset (E) demonstrates double and single stained cells. (F) Double staining for prolactin (TRITC, red colour) and VEGF (FITC, green). Many cells stain for prolactin and VEGF (yellow). VEGF, vascular endothelial growth factor.

**Figure 3:**
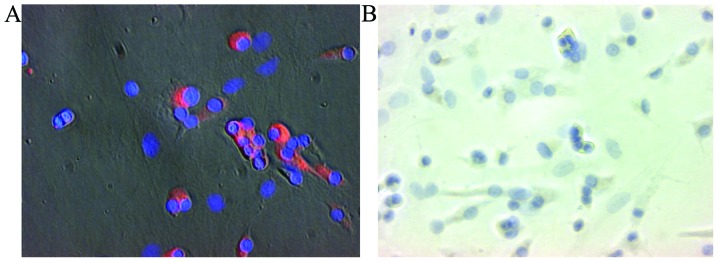
Negative controls. For negative controls phosphate-buffered saline (PBS; Dulbecco), without Ca^2+^ and Mg^2+^ containing 1.5% BSA, replaced the primary antibody. In addition, rabbit immunoglobulin G (IgG; Dako) and goat IgG (Dianova) were used at the identical concentration as the primary antibody. (A) Double immunohistochemistry with rabbit IgG replacing VEGF antibody. Prolactin positive cells are red (TRITC). There is no green signal for VEGF. (B) Goat IgG replacing prolactin antibody. The cells are negative. Negative controls with rabbit or mouse IgG are also negative (data not shown). VEGF, vascular endothelial growth factor; BSA, bovine serum albumin.

Results demonstrate a uniform and nearly identical staining pattern for VEGF and prolactin on paraffin serial sections of pituitary cells (Fig. 2A and B). Detection of lactotroph cells on cytospin-preparations was performed in pituitary cell culture (Fig. 1D). Approximately 50% of the cells stained were positive for prolactin. After seven days of culture, the cells with small nuclei (functional pituitary cells) were positive for VEGF, as well as for prolactin (Fig. 2C and D). Cells with large nuclei (fibroblasts, positive for vimentin, data not shown) were negative for VEGF and prolactin. The staining demonstrate that groups of cells are positive for both prolactin and VEGF (Fig. 2E on Matrigel and F on plastic). Few cells were stained only for VEGF. Nearly 21% of the cells were completely negative for both proteins. The staining pattern of cells cultured on plastic, did not differ from those cultured on Matrigel, although the staining intensity for VEGF was appreciably less in the cells cultured on ECM. Negative control with rabbit IgG, replacing the VEGF antibody in immunocytochemistry with prolactin antibody (Fig. 3A), resulted in a positive signal for prolactin whereas rabbit IgG was negative; the negative control with goat IgG (replacing the prolactin antibody, Fig. 3B) resulted in no visual signal. A biopsy of the human endometrium (mid luteal phase) served as a positive control for VEGF-A (18).

### VEGF production in primary rat pituitary cell culture

**Figure 4:**
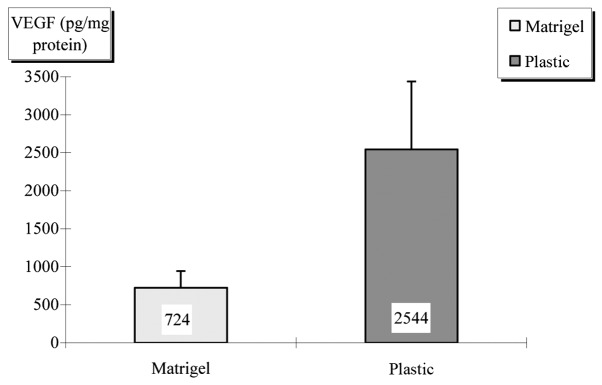
Results of ELISA from culture supernatants. Release of VEGF (pg/mg protein) from primary rat pituitary cells after a 48 h incubation period. ECM [Matrigel-coated wells demonstrate significantly lower VEGF production than uncoated wells (P<0.0022)]. Values are means ± standard deviation. VEGF, vascular endothelial growth factor; ECM, extracellular matrix.

ELISA results reveal less statistically significant VEGF production (P<0.0022) of cells cultured on Matrigel (724±243 pg/mg VEGF, means ± SD vs. cells grown on uncoated plastic wells (2544±951 pg/mg VEGF, means ± SD) (Fig. 4).

## Discussion

This study demonstrates for the first time, since the identification of VEGF in FS (1) that lactotrophs are the main source of VEGF production in the normal pituitary gland.

Jabbour *et al* (11) identified scattered positive FS cells within the sheep pituitary. Approximately 80% of non-FS cells were stained positive for VEGF and remained unidentified. Lactotrophs apparently are the main source of VEGF-production in the pituitary gland and represent a significant portion of the unidentified population.

Here, we demonstrate that FS cells are only infrequently present *in vivo* (1.6%, paraffin section of rat pituitary), after dispersing cells in cell culture (cytospin-preparation) and *in vitro* (2.4%), as identified by two different antibodies against S-100 protein. Our results differ from the data of Vankelecom *et al* (4), where they identified 7.5% FS cells in mouse pituitary using another antibody against the S-100 protein. This can be explained by the fact that the quantity of pituitary FS cells varies between different rat strains such as Fischer 344 and Sprague Dawley rats used in this study. It has been described that Fischer 344 rats have significantly more FS cells (19). Based on studies, that estrogen-induced prolactin secreting tumors in the F344 rat are associated with upregulation of VEGF, it was suggested that lactotrophs may be responsible for VEGF production (20). Our immunohistological detection of widespread VEGF protein signals on cryostat as well as paraffin sections of the anterior pituitary gland confirm the results of Ochoa *et al* (10). They detected a uniform expression VEGF mRNA by *in situ* hybridization throughout the anterior lobe of the rat pituitary gland. This uniform mRNA expression indicates that not only scattered FS cells were responsible for the VEGF mRNA production. It was speculated that lactotrophs and FS cells, which together comprise 50–60% of all pituitary cells (21), may be involved in the VEGF secretion. Our investigations show that lactotrophs are the main source of VEGF production under physiological conditions.

Since curcumin suppresses VEGF release in pituitary adenomas, it has been suspected that it may inhibit pituitary adenoma progression not only through anti-proliferative and pro-apoptotic actions, but also by suppressing pituitary tumor neovascularization (22). Our results strongly support the notion that curcumin has an important impact on lactotrophs and their VEGF production. It is also notable that estradiol stimulates VEGF and interleukin-6 in human lactotroph and lactosomatotroph pituitary adenomas (23). Estrogen administration enhances the expression of proangiogenic factors (e.g., VEGF) in pituitary grafts (24). Indeed, prolactinomas show higher VEGF protein expression compared to nonfunctioning or ACTH- and GH-secreting adenomas (25). The overexpression of vascular endothelial growth factor in pituitary adenomas is associated with extracellular growth and recurrence. Therefore VEGF and its receptors (VEGFR’s) may play an important clinical role in targeted tumor therapy of pituitary tumors (26).

Results of *in vivo* studies cannot exclude the involvement of contributing factors, i.e., cells or ECM molecules. *In vitro* studies performed to examine the effects of VEGF on endothelial cells show that VEGF in the presence of a basal lamina-type ECM specifically induces fenestrations in endothelial cells (27). Other data suggest that Matrigel is necessary to prevent biased functions of cells in culture. It is well documented that epithelial cells dedifferentiate *in vitro* when cultured on plastic (28). Our results demonstrate that lactotrophs are positive for pan-cytokeratin (data not shown), which is typical for epithelial cells, implying that these cells also need ECM for adequate function. With quantification of VEGF in the culture medium by ELISA, it was apparent that Matrigel inhibits VEGF secretion from pituitary cells in culture. As demonstrated by several authors, ECM supports pituitary cell function. Matrigel reduces proliferation and increases prolactin expression of GH3 cells (29). Pituitary cells cultured on plastic produce elevated amounts of VEGF, which is explained by the increased cellular stress that is induced by the artificial plastic surface. Consequently, the cell culture of lactotrophs on ECM better reflects the physiologic conditions compared to uncoated plastic due to disturbed cell function.

In the present study, we demonstrated for the first time, that lactotrophs, which represent ~50% of the anterior pituitary gland cells, are the major source of VEGF production *in vitro* as *in vivo*. This is important for the interpretation of anti-angiogenic treatment and therapeutic response of pituitary tumors treated with anti-VEGF therapy.

ECM is able to influence VEGF release in primary cell culture. We conclude that *in vitro* studies with cell lines and primary cell cultures should be performed on ECM to avoid false results.

## Figures and Tables

**Table I:** Antibodies.

Antibody	Antigen	Dilution	Source
Monoclonal antibodies			
S-100	S-100 protein	1:100	BioGenex, Hamburg, Germany
Polyclonal antibodies			
Prolactin (rabbit)	Prolactin	1:1000	Biotrend, Köln, Germany
VEGF (147) (rabbit)	VEGF	1:30	Santa Cruz Biotechnology, Heidelberg, Germany
S-100 (rabbit)	S-100 protein	1:300	Dako, Hamburg, Germany
Prolactin (M-19) (goat)	Prolactin	1:30	Santa Cruz Biotechnology, Heidelberg, Germany
